# *Miamiensis avidus*, a Novel Scuticociliate Pathogen Isolated and Identified from Cultured Large Yellow Croaker (*Larimichthys crocea*)

**DOI:** 10.3390/pathogens13080618

**Published:** 2024-07-26

**Authors:** Nengfeng Lin, Ying Pan, Zifeng Zhan, Binfu Xu, Hui Gong, Hong Zeng

**Affiliations:** 1 Institute of Biotechnology, Fujian Academy of Agricultural Sciences, Fuzhou 350003, China; linnengfeng@faas.cn (N.L.); panying@faas.cn (Y.P.); xubinfu@faas.cn (B.X.); gonghui@faas.cn (H.G.); 2 Institute of Oceanography, Chinese Academy of Sciences, Qingdao 266071, China; zzhan@qdio.ac.cn; 3 State Key Laboratory of Mariculture Breeding, Ningde 352115, China; 4 The Public Service Platform for Industrialization Development Technology of Marine Biological Medicine and Product of State Oceanic Administration, College of Life Sciences, Fujian Normal University, Fuzhou 350117, China

**Keywords:** *L. crocea*, Scuticociliatosis, *Miamiensis avidus*, α-tubulin indirect immunofluorescence, molecular identification

## Abstract

Scuticociliates are recognized as the causative agents of mass mortalities in certain cultured marine fishes, resulting in enormous economic losses. This study aimed to investigate a fatal infection caused by scuticociliates in farmed large yellow croaker (*Larimichthys crocea*) in Fujian province, China. Microscopic examinations of focal organs, including the brain, eyes, gills, and skin, revealed the presence of parasites. Active masses of scuticociliates were observed in these organs, and the ciliates were subsequently isolated and maintained in vitro. An immersion challenge experiment revealed that *L. crocea* experienced cumulative mortalities reaching 73% within 7 d upon exposure to 1.0 × 10^4^ ciliates mL^−1^. Based on the microscopic and PCR testing of infected fishes, the brain was comprehensively inferred as the main infection organ for the isolated strain. Microscopic and submicroscopic observations of the isolated scuticociliate, coupled with cortical ciliature patterns revealed through α-tubulin indirect immunofluorescence techniques, identified these scuticociliates as *Miamiensis avidus*. The sequencing of two genetic markers (small subunit ribosomal RNA, SSU rRNA and cytochrome c oxidase subunit I, COI) further confirmed that the isolated strains exhibited the highest sequence similarity to most *M. avidus* sequences in GenBank. However, significant differences in SSU sequences compared to the *M. avidus* strain Ma/2, and the lack of published COI and ITS (internal transcribed spacer) sequences for Ma/2, indicate the need for further molecular data to resolve whether there are potential cryptic species within the *M. avidus* complex.

## 1. Introduction

The rapid expansion of fish aquaculture, coupled with increased stocking densities, has resulted in more frequent outbreaks of serious diseases, threatening the sustainable development of the industry. Large yellow croaker (*Larimichthys crocea*) is one of the most economically important marine fish species in China and East Asian countries, with China alone producing 257,683 tons in 2022 [1]. Ciliate pathogens like *Crypotocaryon irritans*, responsible for severe diseases in large yellow croaker aquaculture over the past decade, have received considerable attention [2]. The increase in seedling quantity of large yellow croaker and the change of the mariculture environment have led to recurrent outbreaks of scuticociliatosis in recent years [3,4,5,6].

Some scuticociliates, such as *Uronema marinum*, *Pseudocohnilembus persalinus*, *Philasterides dicentrarchi*, and *Miamiensis avidus*, recognized as histophagous opportunistic parasites, often infect mariculture animals worldwide, causing significant economic losses [7,8,9,10,11,12,13]. The mortality resulting from infections by scuticociliates has been reported in a wide variety of teleost and elasmobranch fish species [14,15].

Although there have been reports of large yellow croakers infected with scuticociliates, in the papers by Zhang (2011) and Zhang (2022) [3,4], the pathogen has not been identified. Other reports only provide a general description of the morphology and molecular identification results [5,6], but they did not include infraciliature descriptions, which are an important basis for the classification of ciliates.

In the present study, we conducted an investigation on scuticociliatosis occurring in cultured juvenile large yellow croakers in Fujian province, China, during the spring season, which ultimately resulted in significant mortality. The diseased fish displayed clinical signs of sluggish swimming, skin ulcerations, anemia, exophthalmos, and skull exposure. Numerous ciliates were observed in the gills, ocular fundus, and brain, but the ciliate pathogen remains unidentified. Consequently, this study aims to identify the ciliates through their infraciliature and related molecular classification markers. Additionally, we intend to assess the pathogenicity of this ciliate, thereby laying a foundation for future research on the prevention and control of scuticociliatosis in large yellow croakers.

## 2. Materials and Methods

### 2.1. Sampling of Large Yellow Croakers and Locality

Samples of large yellow croaker were collected from Sandu Bay (26°37′ N 119°46′ E), Ningde, Fujian province; and Shaceng Bay (27°13′ N 120°23′ E), Fuding, Fujian province. The net cages measured 8 × 8 m, with a water depth of 4 m. The water temperature at the sampling points was 16 ± 2 °C, and the salinity ranged from 28 to 30. Juveniles of large yellow croaker had reached a body length of 40.5 ± 5.9 mm (*n* = 300) and an average weight of 1.24 ± 0.54 g. A total of 20 moribund samples were collected from different cages of the two bays for further analysis.

### 2.2. Sample Collection and Inspection

Superficial mucosa, ulcer tissues, heart blood, and periorbital fluid were smeared and observed under an optical microscope. Wet preparations of the gills, brain, liver, spleen, and metanephros were examined for the presence of ciliates. The morphology of live ciliates was observed and photographed using a Nikon Eclipse 200 microscope (Nikon, Tokyo, Japan).

### 2.3. Ciliate Isolation and Cultivation

A small piece of the brain of a large yellow croaker containing active ciliates was inoculated into filtered seawater (FSW, filtered with 0.22 μm Millipore filter) and incubated at 18 °C. The ciliates were sub-cultured and maintained in 15 mL of FSW with a small piece of cooked shrimp, with inoculation every 5 days. Two isolates from Sandu Bay and Shaceng Bay were named xiapu1 and shaceng1, respectively. Before establishing a monoclonal culture, a 48-well cell culture plate was prepared, with each well containing 500 μL of FSW. The ciliates were appropriately diluted in FSW to achieve a suitable density for the following procedure. Subsequently, a single ciliate was meticulously transferred into each well using an oral pipette under the guidance of a stereomicroscope. An amount of 10 μL of FSW-resuspended *E. coli* DH5α was added to each well. The plate was then incubated at 18 °C. Following a period of 2–3 days, a clone exhibiting robust growth was selected to be established as a monoclonal ciliate strain.

### 2.4. Experimental Infection

Strain shaceng1 was cultured for 3 d while feeding with *E. coli* DH5α, and subsequently centrifuged at 600× *g* for 10 min at 10 °C to collect ciliates. The ciliates were then suspended in FSW, and ciliate numbers were estimated using a plankton counting chamber. Large yellow croakers (mean body length: 50 mm; mean weight: 1.8 g) were kept in an indoor tank for 1 wk prior to infection. A total of 37 fish were exposed to the scuticociliates in 20 L of aerated seawater containing 1.0 × 10^4^ ciliates·mL^−1^. A control group of 20 fish was treated similarly, but without exposure to ciliates. After 8 h, an additional 20 L of seawater was added to the tank. The aquaria were kept in a dimly lit room, and the water temperature was maintained at 17 to 18 °C. During the infection period, no feeding was administered, and continuous aeration was provided. Dead fish were collected in time, and their eyes, brains, gills, skin, and viscera were subjected to molecular biological and histopathological examination.

### 2.5. Molecular Biological Examination

Genomic DNA of the tissue collected from fish was extracted by the FastPure^®^ Tissue isolation Mini Kit (Nanjing Vazyme Biotech Co., Ltd., Nanjing, China). Then, the concentration of each DNA sample was adjusted to 100 ng·μL^−1^. Specific primers of scuticociliates (SSU244-R and SSU1117-F) were using to inspect whether there are scuticociliates in the tissue of dead and moribund fish [5]. PCR amplification system: Premix Taq™ (TAKARA, Dalian, China), 25 μL; DNA, 1 μL; each forward and reverse primer (10 μmol·L^−1^), 2 μL; distilled water, 20 μL. The PCR conditions were as follows: initial denaturation at 94 °C for 3 min, an additional 30 cycles (94 °C for 30 s, 54 °C for 30 s, 72 °C for 1 min), and a final extension at 72 °C for 10 min. PCR products were detected by 1.2% agarose gel electrophoresis.

### 2.6. Histological Examination

Samples were fixed in neutral paraformaldehyde fixed solution (Wuhan servicebio technology Co., Ltd., Wuhan, China) for more than 24 h, dehydrated with gradient alcohol, embedded in paraffin, and sliced into 4 μm sections. The sections were then stained with hematoxylin and eosin. Scanning was performed using Pannoramic DESK (3DHISTECH, Budapest, Hungary). The scanned images were viewed and captured with CaseViewer 2.4.

### 2.7. Preparation of Specimen for Scanning Electron Microscopy (SEM)

The specimen was fixed with 3% glutaraldehyde in PBS (pH 7.0) for more than 4 h, washed three times in PBS, and then fixed with 1% OsO_4_ in PBS (pH 7.0) for 4 h, followed by three additional washes in PBS. Next, the specimen was dehydrated using a graded series of ethanol (50%, 70%, 80%, and 90%) for approximately 10–15 min in each step. Subsequently, the specimen was transferred into 100% ethanol for dehydration three times. The dehydrated sample was treated twice with propylene oxide. Finally, the specimen was dehydrated in a HITACHI critical point dryer (model HCP-2, HITACHI, Tokyo, Japan) with liquid CO_2_. The dehydrated specimen was coated with gold–palladium and photographed on a JEOL (JSM-6380LV) SEM (Japan Electronics Co., Ltd., Tokyo, Japan).

### 2.8. Indirect Immunofluorescence

The indirect immunofluorescence technique was employed to observe the structure of the mouthparts and cilia schema. The method described by Arregui et al. [16] was used with slight modifications as follows: Ciliates were fixed in a 2% paraformaldehyde PHEM (60 mmol·L^−1^ of PIPES, 25 mmol·L^−1^ of HEPES, 10 mmol·L^−1^ of EGTA, 2 mmol·L^−1^ of MgC1; pH = 6.9) solution (dissolved in FSW), and then permeabilized in the extraction buffer with 0.1% triton for 3–5 min. After a quick wash in cold extraction buffer without Triton, cells were rinsed in PBS + 3% bull serum albumin (BSA) buffer three times. The primary antibody (mouse monoclonal anti-α-tubulin, Sigma, St. Louis, MO, USA) was diluted 1000 times and incubated with the cells for 1 h. After washing in the PBS+3%BSA buffer three times, cells were incubated in the secondary antibody (FITC-conjugated Goat Anti-Mouse IgG(H+L), DINGGUO CHANGSHENG biotechnology Co., Ltd., Beijing, China) at a 500-fold dilution for 1 h at room temperature, and then washed three times in the PBS + 3% BSA buffer. After removing the buffer, a drop of antifade mounting medium (Sangon Biotech, Co., Ltd., Shanghai, China) was added. The ciliates were observed and photographed using a Leica confocal microscope (TCS SP8) (Leica, Wetzlar, Germany). The nuclear apparatus was visualized by fluorescence after staining ciliates with an aqueous solution of 0.4 μg·mL^−1^ of 4′−6-diamidine-2-phenylindone (DAPI, Sigma-Aldrich, St. Louis, MO, USA).

### 2.9. Molecular Identification

Ciliates were cultured using sterilized sea water, with *E. coli* DH5α as a food source for 3–4 days at 18 °C. Before genomic DNA extraction, ciliates were treated with lysozyme as described by Xiong et al. [17]. The DNeasy^®^ blood and tissue kit (QIAGEN, Hilden, Germany) was used to extract genomic DNA from approximately 1 × 10^5^ collected ciliates according to the manufacturer’s instructions. The eukaryotic universal primers A (5′-ACCTGGTTGATCCTGCCAGT-3′) and B (5′-TGATCCTTCTGCAGGTTCACCTAC-3′) [18] were used to amplify the small subunit ribosomal RNA (SSU rRNA) gene of the ciliates as described by Jung et al. [19].

Mitochondrial cytochrome c oxidase subunit I (COI) sequences of scuticociliates were downloaded from GenBank. Alignment was performed using MEGA version 11 [20], and the highly conserved region was selected as the 3′ end of the primers. Several degenerate sites were modified to highly homologous bases. Finally, primers Cila-COI-F: 5′-TTACAAGTTATTACCGCACATGG-3′ and Cila-COI-R: 5′-CTATGCCTCAACAGGCATACA-3′ were used for amplification. PCR reaction conditions were as follows: initial denaturation at 94 °C for 5 min, an additional 30 cycles (94 °C for 45 s, 50 °C for 1 min, 72 °C for 1 min), and a final extension at 72 °C for 10 min.

PCR products of the SSU rRNA gene and COI gene were purified via agarose gel electrophoresis and extracted using the SanPrep column DNA gel extraction kit (Sangon Biotech, Co., Ltd., Shanghai, China). Purified PCR products were cloned into the pMD™19-T vector (Takara Biotech, Dalian Co., Ltd., Dalian, China). *E. coli* TOP10 competent cells were used for transformation. Plasmid DNA was extracted by the SanPrep column plasmid mini-prep kit (Sangon Biotech, Co., Ltd., Shanghai, China) and used for sequencing. Two positive clones were selected for sequencing.

Sequencing was performed in both directions with an ABI-PRISM 3730 automatic sequencer (Applied Biosystems, Waltham, MA, USA).

Sequences of the genes were searched online using BLAST (http://www.ncbi.nlm.nih.gov/BLAST/, accessed on 27 May 2024) [21] against the NCBI database to classify the ciliate based on gene similarities (>98%).

Several species of scuticociliates and *Tetrahymena thermophila* were selected to investigate their phylogenetic relationships. The COI and SSU rDNA sequences were derived from GenBank. Sequence alignment was executed using ClustalW 1.8.1. Thereafter, MEGA version 11 [20] was employed to construct the phylogenetic tree utilizing the neighbor-joining (NJ) method. A bootstrap analysis with 1000 replicates was performed to assess the reliability of the phylogenetic tree.

## 3. Results

### 3.1. Clinical Symptoms of Natural Infection

**Figure 1:**
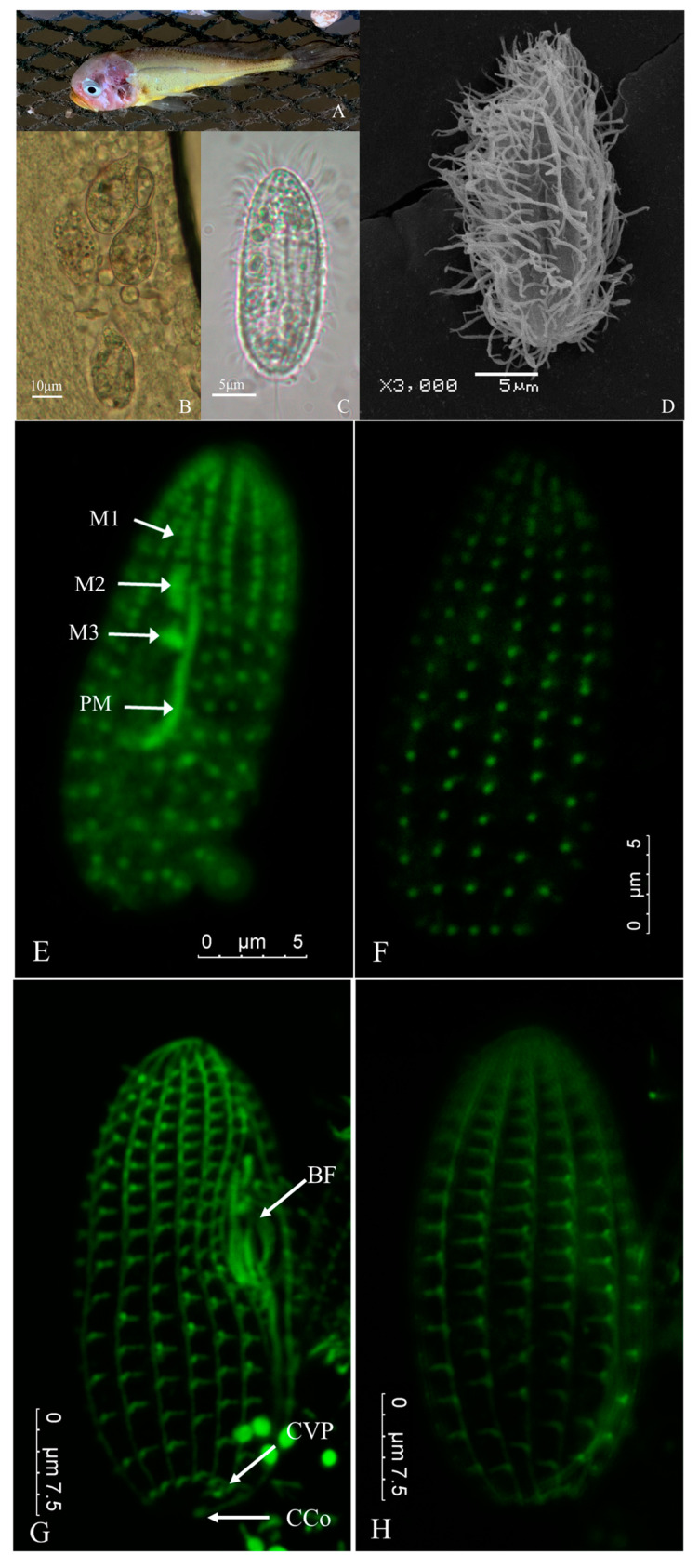
(**A**) Diseased juvenile *Larimichthys crocea*. (**B**) Microscopic morphology of ciliates from wet mount preparations of a skin sample scraped from an ulcer (10 × 40). (**C**) Morphology of ciliates cultured in laboratory conditions (10 × 100). (**D**) External morphology of ciliates observed by scanning electron microscopy (×3000). (**E**) α-tubulin indirect immunofluorescence showed the structure of the cytostome and ventral infraciliature. M1–M3: membranelles 1−3; PM: paroral membrane. (**F**) The dorsal somatic kineties. (**G**) The structure of oral ciliature and caudal cilium complex. BF: buccal field; CVP: contractile vacuole pore; CCo: caudal cilium complex. (**H**) Doral view of G.

Typical signs of naturally infected fish were as follows: slow swimming on the surface of the water; redness of the head due to inflammation; the cerebrum was visible as scales fell off; some fish had ocular proptosis; the branchiostegites were perforated and the gills were severely ulcerated; and there were multiple ulcers on the body surface (Figure 1A). The bodies of the dead fish sank to the bottom of the net. The cumulative fatality rate of the infection could exceed 50%, and almost complete mortality was observed in some cages.

Upon microscopic examination, numerous parasitic ciliates exhibiting similar morphology and movement patterns were observed in the tissues of skin ulcers, brains, and eyes of the diseased fish. However, no ciliates were detected in the blood, ascites, liver, opisthonephros, or spleen.

### 3.2. Morphological Observation of Parasite

The isolated ciliates measured approximately 20–45 μm in length and 15–25 μm in width. In vivo, they exhibited a similar ovoid shape, with a rounded posterior and tapering anterior (Figure 1B). However, ciliates cultured in the laboratory displayed a more slender morphology compared to freshly isolated specimens (Figure 1C). The body surface was densely covered with somatic ciliature (Figure 1C,D), and the cilia in the buccal field were slightly longer than the somatic ciliature. A single caudal cilium measured about 15–20 μm in length. The buccal field was slightly concave and positioned in the middle to fore sections of the body (Figure 1B). Numerous food vacuoles of different sizes filled the cytoplasm and a single contractile vacuole was observed at the posterior end of the soma (Figure 1B,C). One spherical macronucleus was located in the middle of the body (Figure 1 B,C).

### 3.3. Experimental Infection

**Figure 2:**
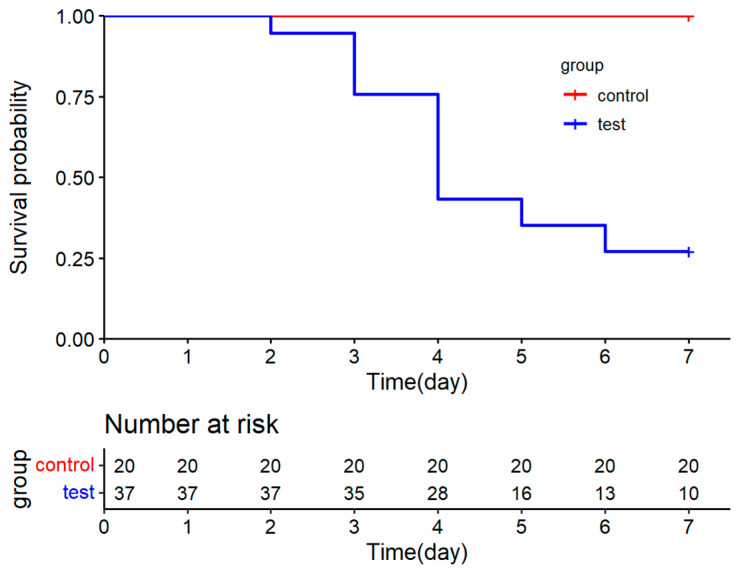
Survival probability curve of *L. crocea* immersion-challenged with *M. avidus* strain shaceng1 at 1 × 10^4^ cell·mL^−1^.

There are some notable differences between the experimentally infected and naturally infected large yellow croaker. No ulcers or hemorrhages were observed around the fins and body. However, the moribund fish that were immersion-challenged with the ciliate exhibited a high number of ciliates in their gills and brain tissue, resembling those observed in naturally infected fish under the light microscopy of wet mount preparations. Fish began to die on the third day post infection at the treatment concentration of 1.0 × 10^4^ mL^−1^. The cumulative mortality in this experiment reached 73% (Figure 2).

### 3.4. Parasitic Infection of Organ Examination Based on PCR

**Figure 3:**
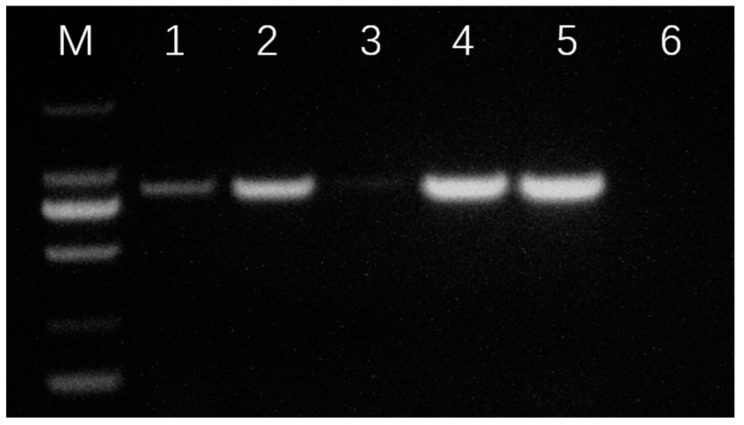
The PCR-based examination of *L. crocea* for ciliate infection. M: DL2000 DNA marker; 1–6: eye, brain, viscera, gills, skin, and negative control (uninfected brain), respectively.

The expected PCR product of the SSU gene fragment was about 870 bp. The samples taken from of the eyes, brain, gills, and skin of infected fish yielded distinct amplification. However, the DNA samples extracted from the viscera only achieved weak amplification (Figure 3).

### 3.5. Histological Examination

**Figure 4:**
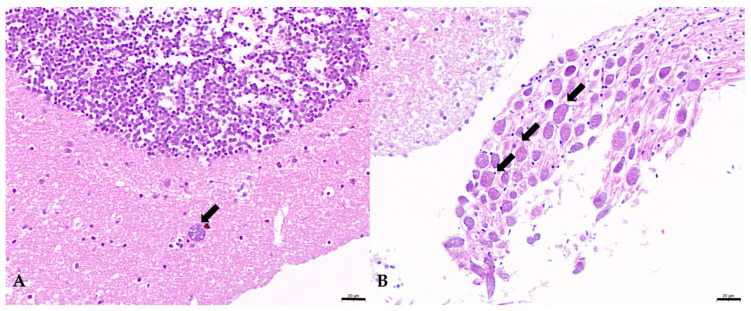
(**A**) Scuticociliates (arrows) inside the brain. (**B**) Scuticociliates (arrows) in the nerve bundles. Brain tissue from infected fish taken on day 3 post infection. Bar = 20 μm.

Under histological examination, ciliates were found predominantly in the central nervous systems of the experimental infected fish. Ciliates containing numerous erythrocytes in the cytoplasm were observed in the brain (Figure 4). Severe gill erosion was observed in naturally infected fish, but not in the experimentally infected groups. Perhaps the most significant pathology in the brain was the presence of ciliates and consequent hemorrhaging.

### 3.6. Indirect Immunofluorescence

The somatic ciliature and buccal apparatus were visualized using the α-tubulin indirect immunofluorescence technique (Figure 1E–H). Somatic kineties (SKs) were longitudinally arranged, maintaining a constant number of 12. Each SK comprised approximately 17–20 kinetoplasts (Figure 1F,H). As depicted in Figure 1E, membranelle 1 (M1) and membranelle 2 (M2) were narrow and relatively long, aligned longitudinally along the anterior portion of the buccal apparatus. They possessed 2 and 4–5 longitudinal rows, respectively. Membranelle 3 (M3) was small, positioned close to M2, and typically consisted of 2–3 rows of kinetosomes. The paroral membrane (PM) originated near the midpoint of M2, curved slightly in the middle, and then followed the curvature of the oral cavity depression. At the posterior end of SK2, a contractile vacuole pore was observed (Figure 1G). Based on external morphology, no significant difference was noted between the two isolates. And the present infraciliature resembled characteristics close to the original descriptions of *Miamiensis avidus* by Thompson and Moewus (1964) [22].

### 3.7. Molecular Identification

The analysis of the similarity in the SSU rRNA gene fragment (1668 bp) revealed that both isolates exhibited identical sequences (Accession Nos. MN611447.1, MN611448.1). Furthermore, these isolates displayed a 99.82% sequence identity with the pathogenic *M. avidus* strain (Accession No. KY082893.1), which was isolated from pharaoh cuttlefish (*Sepia pharaonis*) cultured in Xiangshan Bay, Zhejiang province, China.

The COI gene (676 bp fragment) had only one base difference between the two isolates (Accession Nos. MN688231 and MN688232). The COI gene sequence of xiapu1 demonstrated 100% homology with *M. avidus* strain SJF-03B (Accession No. EU831216.1), which was isolated from olive flounder (*Paralichthys olivaceus*).

**Figure 5:**
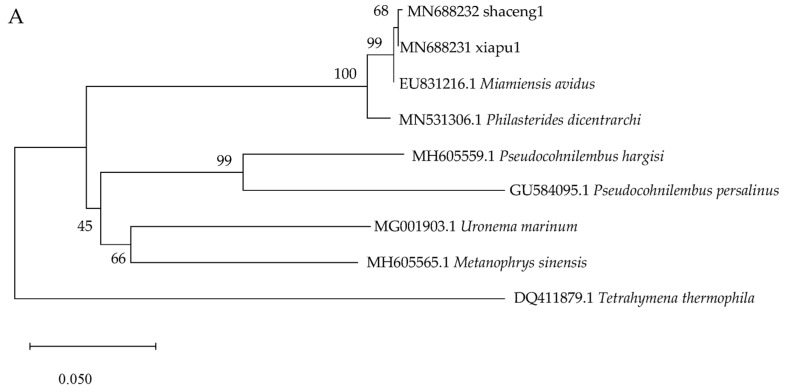

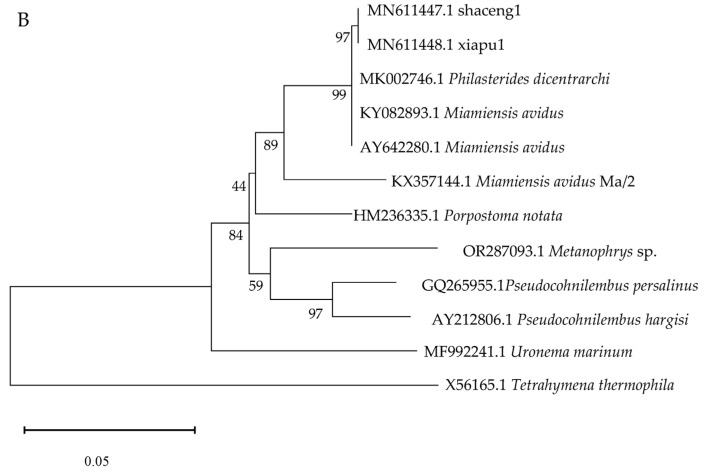
Analysis of the phylogenetic relationships among *Miamiensis avidus* and several ciliate species based on the neighbor-joining method. (**A**) COI (cytochrome c oxidase subunit I gene), (**B**) SSU rDNA (small subunit ribosomal RNA gene).

Phylogenetic analysis revealed that the two isolates examined in this study clustered with *M. avidus*, utilizing both COI and SSU rRNA gene sequences. Meanwhile, both phylogenetic analyses based on COI and SSU rRNA gene consistently showed that *M. avidus* and *Philasterides dicentrachi* cluster together within a single monophyletic group. The phylogenetic tree based on SSU rRNA gene sequences revealed that the *M. avidus* strain MA/2 exhibited differences from other *M. avidus* and *P. dicentrachi*. The neighbor-joining phylogenetic tree based on COI and SSU rRNA gene sequences shown in Figure 5A and Figure 5B, respectively.

## 4. Discussion

Scuticociliates, recognized as facultative parasites of mariculture animals, require suitable conditions to become pathogenic. In the present study, the rapid multiplication and spread of scuticociliates may have been primarily attributed to the high stocking density, obstructed water exchange during the neap tide, and the vulnerability of the juveniles reared in seawater net cages.

Previous studies have established that *M. avidus* can infect flatfish through the intraperitoneal route [23], as well as by immersion [24,25]. Our current research has demonstrated that ciliates isolated from clinically diseased *L. crocea* and subsequently cultured in vitro are capable of successfully invading juvenile *L. crocea* via immersion. The artificial infection experiment further confirms that the scuticociliates we isolated are potent pathogens capable of causing primary infections.

Some researchers believe that the ciliates rapidly invade and proliferate in the skin and gills, subsequently spreading to internal organs, in the absence of any additional pathogens such as secondary bacterial invaders [24]. In present study, we observed that natural and experimental infections of *L. crocea* exhibited some remarkably similar clinical signs; for example, both infected fish displayed abnormal swimming behavior, and microscopic examination revealed a substantial presence of ciliates in the brain. However, the experimentally infected fish that perished did not manifest prominent clinical signs such as ulceration on the body surface or fins. As far as our observation is concerned, the infection of *M. avidus* did not lead to the large-scale infection of internal organs, except for the eyes and brain. PCR analysis and microscopic examination further corroborated that there was no significant presence of ciliates in other internal organs. Moreover, we surmised that the damage and inflammation observed on the body surface under natural infection were attributed to secondary infections. This clinical condition differs from the most prominent clinical signs typically observed in flatfish, which include ulcerations on the body surface and a high concentration of ciliates in the ascites [24,25,26]. Kim et al. [27] examined several organs of olive flounder for *M. avidus* infection using real-time PCR. They also discovered that the brain was one of the first organs to be infected in the early stage. The results of the above study are consistent with our previous hypothesis that the death of large yellow croakers infected with *M. avidus* was primarily caused by the destruction of the respiratory and nervous systems, rather than infection of the viscera [28].

The infraciliature of the two scuticociliates isolates exhibited similar cortical patterns to the original and subsequent descriptions of *M. avidus*, as demonstrated through silver impregnation methods [12,22,24,29,30,31]. It is noteworthy, however, that the *M. avidus* isolated from this study possessed 12 stable somatic kineties, resembling the population described by Zhao et al. [31]. Nevertheless, literature reports vary in the number of somatic kineties, ranging from 10 to 15. For instance, Thompson and Moewus (1964) [22] described the number of longitudinal kineties as ranging from 10 to 13, while Dragesco et al. (1995) [29] reported 13–15 longitudinal kineties, and Jung et al. (2007) [24] documented 13–14 longitudinal kineties. The reasons behind this phenotypic variation remain unknown, and further investigation is necessary to determine whether it represents genuine differences among populations.

The identification of scuticociliates only on the basis of morphological features may lead to misidentification [32]. As previously suggested by DE Felipe et al. [33], a comprehensive approach combining morphological, biological, molecular (via multigene analysis), and serological techniques could enhance the accuracy of identifying scuticociliate parasites in fish. In this study, we employed two genetic markers (SSU rRNA and mtDNA COI) to accurately identify the unknown ciliates. The SSU rRNA gene, a nuclear gene, possesses rich taxonomic characteristics and is highly conserved among species. Therefore, the use of SSU rRNA as a marker for ciliate classification is a universally accepted and highly reliable method [19,34,35]. In contrast, the evolutionary rate of the ciliate COI is exceptionally high and exhibits an unequal rate variation, resulting in a much higher resolution at the intraspecific level. This significant potential allows the COI marker to resolve relationships among closely related ciliate taxa and uncover cryptic species [36]. The molecular identification results obtained in this study were consistent with the morphological identification, further validating the reliability of the molecular identification method.

Although the mitochondrial COI gene exhibits a high evolutionary rate at the intra-specific level, the COI gene sequences of the two isolates differ by only a single base. Additionally, considering the potential migration of parasites with the frequent transactions of large yellow croaker juveniles between the two sampling sites, it is inferred that the two isolates belong to the same strain. Tracking the primary area of onset was challenging, but before the significant mortality of juvenile fishes cultured in net cages, fish hatchery farm workers observed suspected cases of infection with ciliates in some seedling pools. The COI sequence of xiapu1 is identical to that of *M. avidus* SJF-03B, which was isolated from Wando, Korea. According to Jung et al. (2010) [19], SJF-03B can be classified as cox1 type II. The aforementioned findings suggest that *M. avidus* exhibits no significant host selectivity or geographical distribution.

There are considerable debates as to whether *P. dicentrarchi* and *M. avidus* are synonymous [8,24,30,33,37]. The morphology of buccal structures of the specimens displayed by the indirect immunofluorescence technique presented in this study showed that PM1 and PM2 were consecutive, but there is an inward bend near the position of M3, situated between the anterior and posterior sections of the paroral membrane. Hence, we align with the viewpoint put forward by Jung et al. (2007) [24] that the morphology of buccal structures cannot be used as a consistent key for identification of the species. The SSU sequence of the two isolates investigated in this study differed significantly from that of *M. avidus* strain Ma/2. MA et al. [37] considered that there might be cryptic species in *M. avidus*. However, the COI and ITS sequences of *M. avidus* strain Ma/2 have not been published, thus necessitating additional molecular sequence data for thorough analysis. Based on the high similarity of morphology and the high degree of homology in SSU rRNA and COI gene sequence data with most *M. avidus* sequences available in GenBank, the two scuticociliate isolates in this study were tentatively identified as *M. avidus*.

In addition, outbreaks of scuticociliatosis caused by *P. dicentrarchi* tend to occur more commonly during the summer months, when the water temperature is generally higher [11,38,39]. In many reported cases of *M. avidus* infection, a water temperature range of 18−23 °C seems to be optimal for the proliferation of *M. avidus* [12,40,41]. However, the water temperature during the outbreak of *M. avidus* infection in large yellow croaker was 14–18 °C in the present study. It is suggested that the infection caused by *M. avidus* is not overly reliant on water temperature but is more directly associated with the susceptibility of the host.

Recently, scholars have founded instances of *L. crocea* being infected with scuticociliates such as *Porpostoma notata* [5] and *Metanophrys* sp. [6], including the case studied here, where *L. crocea* was infected with *M. avidus*. This collective evidence indicates that *L. crocea* is a susceptible host for scuticociliates. Hence, it is of great significance to intensify research into the mechanisms of scuticociliates infection in *L. crocea*, as well as explore preventative and control measures, in order to safeguard the healthy and sustainable development of the *L. crocea* aquaculture industry.

## Data Availability

The datasets used and/or analyzed during the current study are available from the corresponding author on reasonable request.
